# Feasibility, Perceived Impact, and Acceptability of a Socially Assistive Robot to Support Emotion Regulation With Highly Anxious University Students: Mixed Methods Open Trial

**DOI:** 10.2196/46826

**Published:** 2023-10-31

**Authors:** A Jess Williams, Maureen Freed, Nikki Theofanopoulou, Claudia Daudén Roquet, Predrag Klasnja, James Gross, Jessica Schleider, Petr Slovak

**Affiliations:** 1 Department of Informatics King's College London London United Kingdom; 2 Psychodynamic Studies University of Oxford Oxford United Kingdom; 3 School of Information University of Michigan Ann Arbor, MI United States; 4 Psychophysiology Laboratory University of Stanford Stanford, CA United States; 5 Department of Psychology Stony Brook University New York, NY United States

**Keywords:** emotion regulation, students, anxiety, digital intervention, mixed-methods

## Abstract

**Background:**

Mental health difficulties among university students have been rising rapidly over the last decade, and the demand for university mental health services commonly far exceeds available resources. Digital interventions are seen as one potential solution to these challenges. However, as in other mental health contexts, digital programs often face low engagement and uptake, and the field lacks usable, engaging, evidence-supported mental health interventions that may be used flexibly when students need them most.

**Objective:**

The aim of this study is to investigate the feasibility and acceptability of a new, in situ intervention tool (Purrble) among university students experiencing anxiety. As an intervention, Purrble was designed to provide in situ support for emotion regulation (ER)—a well-known transdiagnostic construct—directly in the moments when individuals are facing emotionally challenging situations. A secondary aim is to consider the perceived impact of Purrble on youth mental health, as reported by students over a 7-week deployment.

**Methods:**

A mixed methods open trial was conducted with 78 under- and postgraduate students at Oxford University. Participants were recruited based on moderate to high levels of anxiety measured by Generalized Anxiety Disorder-7 at baseline (mean 16.09, SD 3.03). All participants had access to Purrble for 7 weeks during the spring term with data on their perceived anxiety, emotion dysregulation, ER self-efficacy, and engagement with the intervention collected at baseline (pre), week 4 (mid), and week 8 (postintervention). Qualitative responses were also collected at the mid- and postintervention points.

**Results:**

The findings demonstrated a sustained engagement with Purrble over the 7-week period, with the acceptability further supported by the qualitative data indicating that students accepted Purrble and that Purrble was well-integrated into their daily routines. Exploratory quantitative data analysis indicated that Purrble was associated with reductions in student anxiety (dz=0.96, 95% CI 0.62-1.29) and emotion dysregulation (dz=0.69, 95% CI 0.38-0.99), and with an increase in ER self-efficacy (dz=–0.56, 95% CI –0.86 to –0.26).

**Conclusions:**

This is the first trial of a simple physical intervention that aims to provide ongoing ER support to university students. Both quantitative and qualitative data suggest that Purrble is an acceptable and feasible intervention among students, the engagement with which can be sustained at a stable level across a 7-week period while retaining a perceived benefit for those who use it (n=32, 61% of our sample). The consistency of use is particularly promising given that there was no clinician engagement or further support provided beyond Purrble being delivered to the students. These results show promise for an innovative intervention model, which could be complementary to the existing interventions.

## Introduction

### Background

Globally, the prevalence of mental health difficulties among students is a major concern [1,2]. Recent epidemiological studies estimate that about one-fifth (20.3%) of all university students are affected by mental health disorders every year [1,3,4] with the incidence rates rising substantially over the last 10 years [5]. In particular, anxiety disorders have been highlighted as a concern among student populations; for example, a recent review across 21 countries shows 11.7%-14.7% of the cross-national population being impacted [1]. In recent years, this concern has grown as generalized anxiety disorder alone is now thought to be affecting 16.7%-18.6% of students over 12 months to lifetime prevalence [3]. Alongside anxiety disorders, students are known to struggle with mood and substance use disorders [1-3] that can be associated with the onset of university education [1].

Traditional university services have struggled to meet the increasing needs of this population [6-8], with student demand for mental health services far exceeding changes in enrollment numbers [9,10]. Combined with difficulties such as underresourced counseling centers and lack of staff capacity, students often face extended waiting times when seeking help [5,11]. This may further exacerbate symptoms of mental ill-health and discourage future help-seeking [12].

### Digital Mental Health Interventions for University Students

One potential solution to these challenges involves the use of digital technologies that are generally regarded positively by university students [13]. Prior work has shown that web-based service provision can be efficacious with a recent systematic review [14] showing small intervention effects on anxiety (Hedges *g*=0.27), depression (*g*=0.18), and stress (*g*=0.20) in randomized controlled trials, amid substantial heterogeneity and inclusion of high risk of bias studies in the sample. The 48 intervention programs reviewed were typically delivered through websites (89.6%), focused on cognitive behavioral therapy (or third-wave cognitive behavioral therapy) skills training (66%), and relied on a passive control group (eg, waitlist or psychoeducation materials for behavior change) [14]. We note, however, that these effects are smaller than those seen in similar systematic reviews of interventions for general populations (eg, for digital anxiety treatments, *g*=0.62 [15]).

Moreover, the implementation of digital programs within routine services remains challenging in student populations [16-18]. Pragmatic trials on web-based interventions show low engagement and uptake rates [19-22] similar to web-based mental health interventions in other domains [23]. To overcome such a lack of participant engagement, there is a need to develop usable, engaging, evidence-supported mental health interventions that may be used flexibly based on when students need them most (eg, when stress levels are particularly high and coping skills most warrant deployment).

### Emotion Regulation and Emerging Digital Interventions

Emotion regulation (ER) is a well-known transdiagnostic factor for a range of mental health difficulties [24-27]. Across young people, difficulties with ER have been associated with a range of mental health difficulties, such as eating disorders [28], anxiety and depression [29], as well as self-harm and suicidal thoughts [30]. In student populations particularly, difficulties with ER have been found to be moderately severe [31,32], with dysregulation related to practices such as maladaptive perfectionism [33] and coping [34].

While the research on digitally mediated interventions targeting ER is limited to date (cf, recent review [35]), there is one emerging intervention tool—“Purrble”—which was designed to provide in-the-moment ER support in daily life [24,36,37] (see Intervention section for more details). Purrble was originally developed for use with child populations, and there is emerging evidence that it could deliver significant benefits through improved ER [24,36,37]. For example, Purrble has been shown to facilitate positive parental-child interactions [24,36], support children to self-soothe in the moment [24,37], and enable conversations around children’s emotions [37]. Within interviews, parents and children recognized that Purrble was incorporated into perceived ER practices (such as disengagement or distraction), which helped to calm or settle the child in emotion-eliciting situations [38]; the children also remained engaged with Purrble across the studies [24,37,38]. From a theory-of-change perspective, Purrble is based on the Gross Process model of ER [39]: it has been designed to impact two separate stages of the ER process, (1) the attention deployment stage, whereby an individual’s attention is shifted from the emotion-eliciting situations toward interacting with the toy; and (2) the response modulation stage, by facilitating downregulation through pleasant tactile interactions—see prior work [36] for further details.

In summary, while the emerging evidence—together with the theory of change—suggests that Purrble could support ER across a broader age range, no work has so far investigated if and how older populations such as university students might engage with Purrble during stressful periods (eg, during the college term) and how this may impact prevalent mental health concerns, such as anxiety.

### This Study

The aim of this feasibility and acceptability study is to evaluate a novel digital intervention device, Purrble, designed to provide in-the-moment ER support in daily life [24] with high-anxiety students in naturalistic contexts. Therefore, the specific objectives are to determine the feasibility of Purrble as an emotion regulatory intervention by considering retention rate, engagement measures, and perceived impact on mental health; to assess the acceptability of Purrble by exploring students’ perceptions and experiences of engaging and appropriating the device using the open-text responses to mid- and postdeployment surveys; and to determine the association between Purrble deployment and students’ anxiety symptoms, ER difficulties, and ER beliefs.

## Methods

This study is an open trial deployment of an in situ intervention to enhance in-the-moment ER in high-anxiety university students.

### Ethics Approval

Ethics approval was obtained from the University of Oxford ethics board (CUREC: 310221) prior to the start of the investigation.

### Recruitment

Participants were recruited using advertisements in a university-wide newsletter that included a link to register interest in the study. To take part, interested individuals completed a short screening tool made of a battery of validated measures. To be eligible, one needed to be (1) an Oxford University student, (2) 18 years or older, (3) living in Oxford at the start of the study, and (4) score 10 or above on the Generalized Anxiety Disorder-7 (GAD-7), indicating moderate to severe anxiety symptoms. Due to the limited number of intervention devices available, additional criteria were needed for participant selection, offering priority to nonbinary or male participants due to the high volume of interest from cisgender female students and those with severe anxiety. All selected eligible students were sent consent forms to complete.

### Intervention

**Figure 1:**
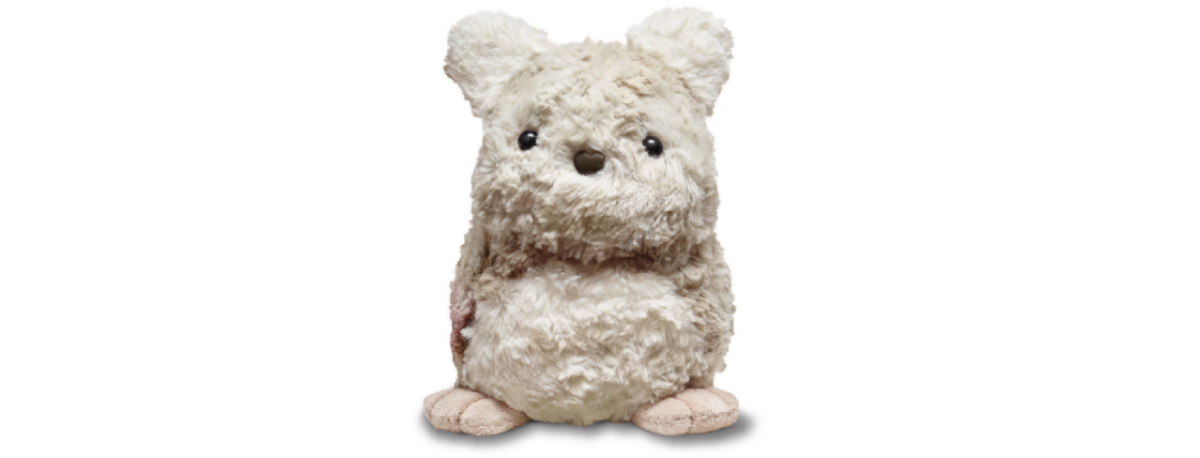
Intervention tool—Purrble.

Purrble is a small, inexpensive, web-based plush animal (Figure 1) that aims to guide the user to downregulate their unwanted emotions through a combination of sensors and haptic vibrations, with the theory-of-change targeting the attentional deployment and response modulation components of the ER process [24,25]. The smart toy’s internal state is communicated to users through vibration patterns that mimic a heartbeat, with faster rates corresponding to higher “stress” levels. The fundamental game loop is the following: whenever the smart toy wakes up from sleep, it is startled and has a rapid heartbeat. Shakes, sudden movements, or pressing its ears also wake and “startle” the toy, while calm stroking movements and hugs gradually slow the heartbeat, which eventually changes to a purring vibration, indicating a calm, happy state. The toy also makes varying gentle sounds (ie, sighs, coos, giggles, and grunts) that correspond to its internal state and complement the vibration-based feedback. The full description of the intervention including the detailed theory-of-change model is available [36].

What makes Purrble a potentially unique intervention mechanism is the combination of (1) physical form, (2) associated ongoing availability to provide support as and when needed in everyday settings, and (3) the targeting of the process of in-the-moment ER. Such ongoing in-the-moment ER support—if effective—has the potential to create a positive feedback loop and drive further engagement: for example, if a student is able to reduce their anxiety by interacting with Purrble (and thus feels better), it is more likely that Purrble will become embedded into their daily ER practice. In addition, the in-the-moment physical intervention delivery aims to remove the need for any a priori training on the part of the student and makes Purrble an interestingly complementary approach to existing cognitive interventions (which are reliant on training modules while lacking in-the-moment support for skills application).

### Measures

#### Demographics

Information, such as university, college, student status, location, and gender, was collected as part of the screening process. These were used to ensure inclusion criteria were met.

#### Feasibility Measures

Digital engagement of Purrble was assessed using Twente Engagement With eHealth Technologies Scale (TWEETS) [40], which consists of 9 items measured on a 5-point Likert scale ranging from strongly disagree (0) to strongly agree (4), with higher scores indicating greater engagement. This measure has been shown to have good reliability, in this sample, Cronbach α was .88.

Within the postdeployment survey, participants were asked whether they felt Purrble had helped or hurt their mental health using a 5-point Likert scale of strongly disagree (1) to strongly agree (5). This provided a bespoke measure of Purrble’s perceived impact on their own mental health.

#### Acceptability Measures

Exploratory open-ended questions were included in the mid- and postintervention surveys. These queried students about situations where Purrble was used, why or why not they used Purrble, perceived emotional impact, usefulness, and personal ER strategies.

#### Mental Health Measures

##### Generalized Anxiety Disorder-7

The GAD-7 [41] is a 7-item screening tool designed to assess the presence and severity of generalized anxiety disorder. Individuals are required to rate the frequency of anxiety symptoms on a 4-point Likert scale from not at all (0) to nearly every day (3). Higher scores indicate greater severity of anxiety symptoms, with a threshold of 10 suggesting moderate and 15 implying severe anxiety. In this sample, Cronbach α was .88.

##### Difficulties in Emotion Regulation Scale-18

The Difficulties in Emotion Regulation Scale-18 (DERS-18) [42] is an 18-item questionnaire that assesses how well participants regulate their emotions. Participants are asked to rate how often each statement applies to them on a 5-point Likert scale ranging from almost never (1) to almost always (5). Higher total scores imply greater difficulties with ER. In this sample, Cronbach α was .91.

##### Beliefs About Emotion (ER Beliefs)

Following the adaptation of the Implicit Beliefs About Emotions scale [43,44], ER beliefs was used as a 4-item measure to assess personal beliefs about the malleability of emotions. Participants are asked to score these statements using a 5-point Likert scale ranging from strongly disagree (1) to strongly agree (5). Those with higher scores are thought to hold fixed beliefs about ER, whereas lower scores suggest more malleable beliefs that may be changed. In this sample, Cronbach α was .83.

### Procedure

Assessments (overview in Table 1) were conducted on the web using King’s College London Qualtrics, with the baseline assessment (anxiety and ER items) taking place the week prior to Purrble deployment and feasibility, acceptability, and mental health measures asked in mid- and postdeployment surveys. Across a 7-week deployment period, participants had access to their individual Purrble with the instructions to use it “as much or as little as they liked.” This was to ensure that any engagement was unbiased and offered an understanding of how students appropriated Purrble over time. Optional daily surveys were sent to participants each evening (~5 minutes), asking about their day and Purrble engagement (Multimedia Appendix 1). Other than the bespoke feasibility measure of impact, all feasibility, acceptability, and mental health measures were asked in both mid- and postdeployment surveys.

Following the intervention period, participants were invited to a semistructured interview, which took place via Zoom (Zoom Video Communications). This was to explore their perceptions and experiences of having had Purrble, whether they had engaged with the intervention, and their understanding as to whether Purrble had influenced their ER skills at all.

**Table 1:** Data collection procedure across the study.

Survey	Predeployment	Deployment								Follow-up
	Week 0	Week 1	Week 2	Week 3	Week 4	Week 5	Week 6	Week 7	Week 8	
Baseline assessment (GAD-7^a^, DERS-18^b^, ER^c^ beliefs)	✓									
Daily surveys		—^d^	—^d^	—^d^	—^d^	—^d^	—^d^	—^d^		
Middeployment survey (GAD-7, DERS-18, ER beliefs, TWEETS^e^, exploratory open-ended questions)					✓					
Postdeployment survey (GAD-7, DERS-18, ER beliefs, TWEETS, exploratory open-ended questions)									✓	
Interviews with a subset of participants									✓	

^a^GAD-7: Generalized Anxiety Disorder-7.

^b^DERS-18: Difficulties in Emotion Regulation Scale-18.

^c^ER: emotion regulation.

^d^Collected daily.

^e^TWEETS: Twente Engagement With eHealth Technologies Scale.

### Mixed Methods Analytic Plan

Statistical data were analyzed using RStudio (Posit) and the *lmer* package. For baseline measures, averages and SDs were calculated to give an insight into the characteristics of the participant sample.

Intervention feasibility was assessed in three ways by calculating descriptive statistics: (1) calculating retention rate across the study period as percentages of students who submitted mid- or postquestionnaires, (2) self-reported engagement with the intervention through a total score of the TWEETS measure for those retained in the study, and (3) perceived impact of intervention as a percentage of student responses at postdeployment survey. The daily items asking about Purrble engagement were not analyzed due to their optional status and thus high attrition (>70% by week 8 of the study).

To assess the acceptability of Purrble, iterative deductive thematic analysis was used to explore the perceptions and experiences of engaging and appropriating Purrble, as per their own needs. This was achieved by considering participant responses to open-ended questions collected during the exploratory surveys. The qualitative data were analyzed following the 6 phases outlined by Braun and Clarke [45,46]. Following survey data cleaning, all data were imported into NVivo (version 12; Lumivero), and AJW began deductively coding the data. Codes were clustered to develop a meaningful preliminary framework, which was discussed with PS. Additional considerations and reflections were discussed, leading to a revised thematic framework. This was reviewed and finalized by all authors. The semistructured interviews of this study are analyzed elsewhere [47].

To explore the association between Purrble deployment and students’ anxiety symptoms (GAD-7), ER difficulties (DERS-18), and ER beliefs, each of the outcome variables was fitted to progressively more complex linear mixed models to identify the best fit, following the analysis steps from prior analogous work [48].

The starting model predicted the outcome variable as a function of time and allowed for both random slopes and intercept for individual participants (unless this reduced the model fit): <outcome> ~ timepoint + (timepoint | participant). We then included predetermined covariates, that is, engagement (TWEETS) and gender. For DERS-18 and ER beliefs, we also included baseline GAD-7 as another covariate. Restricted maximum likelihood methods were used for estimation (the default approach within lme4), which yields unbiased estimates under the assumption that the missing data mechanism is ignorable (ie, data are missing at random [49]). Given the promising trajectory for all variables, within-group effect sizes (Cohen *d*; 95% CI) were calculated to reflect this change from pre- (week 0) to postdeployment (week 7) on each of the outcome variables.

## Results

### Sample Characteristics

In total, 80 (42 undergraduates, 36 postgraduates) students were enrolled in the study. Two participants did not return signed consent forms and were excluded. Of the full sample (n=78), most participants identified as female (n=48). The remaining participants identified as male (n=18) and nonbinary (n=10). Two participants did not list their gender identity. The average age was 23.5 (range 18-40) years.

At the start of the trial, participants self-reported having severe anxiety (mean 16.09, SD 3.03) alongside high scores of emotion dysregulation (mean 55.95, SD 12.76) and lower levels of beliefs that one can control their ER abilities (mean 11.24, SD 3.21). Between genders, symptoms of anxiety were higher among females (mean 18.0, SD 1.75) when compared to male (mean 14.3, SD 2.56) and nonbinary (mean 14.2, SD 2.90) counterparts.

### Feasibility

In total, 300 students responded to the study call. As there was a limited number of Purrble units, 80 of the 142 students who met all inclusion criteria were invited to take part based on the additional criteria. In total, 78 participants provided valid consent forms and completed the baseline assessment; therefore, the enrollment rate was 97.5%. Of the 78 students enrolled, 57 responded to the midpoint assessment (week 4), which decreased to 52 respondents at postdeployment (week 8). Therefore, the overall retention rate across the 7-week deployment including follow-up was 66.7%. We do not see differences in baseline GAD-7 scores between those who dropped out (mean 15.9, SD 3.24; n=25) and those who remained in the study (mean 16.2, SD 2.94; n=51). However, there is some indication that female participants were less likely to drop out (6 out of 28) than male participants (13 out of 32) and nonbinary participants (3 out of 10); it is possible that these differences could be linked to the lower baseline levels of anxiety for male and nonbinary participants.

Across the 7-week deployment, self-reported engagement was measured at the midpoint (week 4) and postdeployment (week 8) through TWEETS. At both time points, students reported a moderate level of engagement with the intervention, with a limited difference from week 4 (mean 23.75, SD 5.09) to week 8 (mean 21.79, SD 6.79). Students who responded to these surveys maintained a good degree of engagement with Purrble throughout the deployment.

Purrble was indicated as a feasible intervention, as generally students reported it to have had a positive impact on their mental health. Of those students who remained in the study, 61% (n=32) reported that Purrble had helped their mental health, while only 9% (n=5) disagreed with this statement. Similarly, 93% (n=48) stated that they “strongly disagreed” or “disagreed” with the statement that Purrble had hurt their mental health, and the remaining 7% (n=4) answered neutrally.

### Acceptability

#### Overview

To determine Purrble acceptability as a tool in students’ daily lives, the qualitative responses from the midpoint and posttrial surveys were explored considering the engagement and appropriation of the intervention. Four themes were developed: (1) primary uses for Purrble, (2) appropriation mechanisms, (3) Purrble is “just” a toy, and (4) empathetic responses (Table 2). The themes identified are described later with example quotes. Additional illustrative quotes by subtheme can be found in Multimedia Appendix 2.

**Table 2:** Thematic framework including the prevalence of instance of subtheme.

Theme and subtheme		Values, n (%)
**Primary uses for Purrble**		
	Tool for anxiety and stress reduction	66 (60.6)
	Calming during night routine	27 (24.8)
	Prevention of mental health spiral	6 (5.5)
**Appropriation mechanisms**		
	Grounding	25 (22.9)
	Mindfulness	13 (11.9)
	Self-stimulation	30 (27.5)
**Purrble is “just” a toy**		
	Feelings of embarrassment	11 (10.1)
	“If levels are too high”	15 (13.8)
	Purrble-related anxiety	21 (19.3)
**Empathetic response**		
	Caring for something else helps one’s ability to self-soothe	32 (29.4)
	Guilt relating to “upsetting” Purrble	8 (7.3)

#### Primary Uses for Purrble

The most frequently reported use for Purrble by students was as a tool for anxiety and stress reduction. The students described using Purrble as a means to cope with anxiety or anxiety-inducing situations, with some indicating specific types of anxiety responses that Purrble was particularly useful for; for example, “Purrble is useful when I have a quality of anxiety that is panicky—by which I mean, heart palpitations and shakes, where my physiological response is in itself problematic” (R_339Y). Purrble was also often incorporated into a calming night routine, either acting as a device to replace negative sleep hygiene practices (“something to focus on that is not a screen, before I go to bed” [R_OveS]) or as a comforting object while sleeping. For a few people, Purrble was used to prevent a mental health spiral, supporting students in stopping their experiences of poor mental health from becoming more severe and difficult to cope with. Purrble was described as being particularly useful to act as a tool to “snap out of the spiraling nature of invasive thoughts” (R_2EGJ).

Together, these descriptions show students’ perceptions of Purrble as an acceptable and effective intervention for anxiety and stress as well as highlight it as a potential mechanism to break the ruminative aspect of mental health difficulties.

#### Appropriation Mechanism

The students also described a number of mechanisms that led to the positive effects stated earlier. Most commonly, students reported using Purrble as a tangible grounding tool—something that allowed them to center themselves when experiencing specific stressors and thus reduce their anxiety: “when I'm dissociating, I use the Purrble to ground myself” (R_1jfX). Purrble was also used alongside mindfulness processes and acted as a physical reminder and a practical tool to bring them to that mindfulness headspace. Finally, Purrble also seemed to support self-stimulation when anxious, allowing students to relax or calm down through touching or stroking the device: “The action of stroking him rhythmically is very calming, and takes the focus away from the source of panic/stress” (R_25vd).

All of these strategies can be linked to specific ER processes (attention deployment, cognitive change, and response modulation), suggesting that Purrble could have the intended impact of supporting in situ ER.

#### Purrble Is “Just” a Toy

While most students felt Purrble was helpful to them, some also had reservations about situations where Purrble could be used. This was commonly associated with feelings of embarrassment due to Purrble being perceived as designed for children and therefore was not always thought to be “appropriate” for adult use in public due to assumed judgment or stigma from others.

A small subset of students also felt that Purrble was not an acceptable option if their levels of distress “are too high,” either as it would not help (“knowing that it is merely a substitute and not an actual living being” [R_3Rz4]) or because it would be too difficult to engage (“it is hard to make myself reach out for him” [R_10ZQ]). Finally, for a few students, there were moments when Purrble interaction could heighten the student’s sensory perceptions, leading to Purrble-related anxiety (“the rare/occasional times where Purrble's heartbeat/sounds trigger my overstimulation/anxiety” [R_28Z2]).

#### Empathetic Response

Across students, Purrble appeared to induce empathetic responses: the participants sought to comfort Purrble when it was “distressed.” The “need” and ability to care for something else helped self-soothing: it allowed the students to detach themselves from their distress and promoted a focus on Purrble, which encouraged students to relax. “It made me feel quite parental and allowed me to get out of my own head for a minute” (R_2Cba). However, the same empathic response sometimes induced feelings of guilt relating to “upsetting” Purrble by waking it to start an interaction.

### Exploring Associations of Purrble Deployment and Mental Health Measures

Findings indicate consistent and statistically significant reductions in all 3 outcome measures when comparing baseline with midterm (week 3) and end-of-term (week 7) observations (Tables 3 and 4). Additionally, these improvements over time are consistent across all models regardless of whether correlates such as TWEETS or GAD-7 baseline are factored in.

First, there were large reductions of anxiety (dz=0.96, 95% CI 0.62-1.29 and dav=1.22, 95% CI 0.85-1.57) with post–week 7 anxiety showing an 83% chance of being lower than baseline. Second, the results show medium changes to difficulties with ER (dz=0.69, 95% CI 0.38-0.99 and dav=0.56, 95% CI 0.26-0.85) with postdeployment scores showing a 75% chance of being lower than baseline. Finally, the outcomes showed medium changes to ER beliefs (dz=–0.56, 95% CI –0.86 to –0.26 and dav=–0.51, 95% CI –0.80 to –0.21) with postdeployment showing a 71% chance of being lower than baseline difficulties with ER.

Midpoint engagement score (TWEETS) was not significantly associated with a reduction in anxiety or difficulties with ER scores but was associated with ER beliefs outcomes (with higher TWEETs at baseline leading to a stronger increase in ER beliefs). In contrast, higher baseline anxiety was associated with a lower reduction in difficulties in ER but not with changes in ER beliefs.

**Table 3:** Results of mixed-linear models considering the impact of Purrble on anxiety symptoms.

			Dependent variable: generalized anxiety disorder
Mid–time point			–4.18^a^ (0.63)
Post–time point			–5.38^a^ (0.67)
TWEETS^b^: mid–time point			–0.10 (0.09)
**Gender**			
	Nonbinary	–0.45 (1.48)	
	Not listed	3.44 (1.76)	
	Female	2.22^c^ (1.06)	
Constant			17.64^a^ (2.44)
Observations			159
Log likelihood			–441.71
Akaike information criterion			901.41
Bayesian information criterion			929.03

^a^*P*<.001.

^b^TWEETS: Twente Engagement With eHealth Technologies Scale.

^c^*P*<.05.

**Table 4:** Results of mixed-linear model considering the impact of Purrble on emotion regulation.

	Dependent variable	
	Difficulties in Emotion Regulation	Beliefs About Emotion
Mid–time point	–3.42^a^ (1.32)	1.31^b^ (0.36)
Post–time point	–7.56^b^ (1.41)	1.59^b^ (0.38)
TWEETS^c^: mid–time point	–0.51 (0.30)	0.235^b^ (0.07)
GAD-7^d^: baseline	1.69^a^ (0.52)	–0.05 (0.12)
Constant	41.39^b^ (10.95)	6.03^e^ (2.59)
Observations	159	159
Log likelihood	–584.41	–375.42
Akaike information criterion	1182.81	764.83
Bayesian information criterion	1204.29	786.32

^a^*P*<.01.

^b^*P*<.001.

^c^TWEETS: Twente Engagement With eHealth Technologies Scale.

^d^GAD-7: Generalized Anxiety Disorder-7.

^e^*P*<.05.

## Discussion

### Principal Findings

This study aimed to determine whether a socially assistive robot, Purrble, would be a feasible and acceptable intervention for highly anxious university students. The overall findings support the feasibility of Purrble and indicate that Purrble was acceptable as an intervention within this sample, offering insights into how students engaged and appropriated Purrble to suit their individual needs. Furthermore, the explorative analysis showed positive within-subject associations of Purrble deployment with reductions in students’ self-reported anxiety symptoms and with the perceived improvement of ER.

Around one-third of the student sample disengaged by week 4 of this study, but those who were retained used Purrble consistently across the trial period, a finding consistent across questionnaires, open-text responses, and qualitative interview data [47]. The overall number of retained participants was comparable to other studies [50-52]. Interestingly, Purrble maintained a stable level of engagement among those retained—as measured by TWEETS score—throughout the deployment with only a minor (and statistically nonsignificant) decrease in use over the 7-week deployment. This is surprising as prior research shows that engagement with digital interventions tends to drop off markedly over time [18,53,54], and ongoing engagement is a key challenge in traditional therapeutic approaches [55]. For example, prior work has reported decreasing rates of app use each week [50], decreasing rates of program adherence over time [51], and only 10.1% of youths completing all full 10 cognitive behavioral therapy sessions [52]. The consistency of Purrble engagement is particularly striking given the lack of any therapist engagement (eg, clinician’s calls) at any point during the intervention that would often be deployed to encourage sustained engagement [56]. This supports emerging arguments about the benefits of interventions that draw on extensive user-centered design approaches and focus on addressing the day-to-day needs of target populations [18,57,58]. In summary, both quantitative and qualitative data suggest that Purrble is an acceptable and feasible intervention among students and can sustain a stable-level engagement across a 7-week period with acceptable dropouts while retaining a perceived benefit for those who use it.

Our exploratory analysis also shows large within-subject effects on student anxiety in week 7 (*d*=0.96), which are akin to results from other recent web-based open trials [52,59-61]. Moreover, the results also show medium effect sizes on the 2 ER constructs: beliefs about own ER self-efficacy (dz=–0.56) and difficulties with ER (dz=–0.69). This provides support for the theory-of-change underpinning Purrble, assuming that repeated in-the-moment downregulation experiences can both improve perceived self-efficacy and mindsets about ER as well as help scaffold more constructive ER practices [47]. We note that what is particularly interesting in this work is that we have observed both changes in the proximal ER constructs and in anxiety even though Purrble intervention does not explicitly contain any cognitive components (eg, cognitive reappraisal or exposure hierarchies), which underpin traditional ER interventions for mood disorders (eg, Unified Protocol [62,63] or ER therapy [64,65]). This suggests that the effects emerging from in-the-moment experiences of Purrble use could be complementary to the traditionally more cognitively oriented interventions with the potential for mutual amplification (eg, where experiential effects of Purrble further support cognitive training and vice versa).

### Implications

Given the promising results found from this trial, implications for wider research are considered. First, given the large effect sizes seen in this open trial, it would be prudent to examine Purrble alongside a control treatment within a randomized controlled trial to determine whether Purrble is indeed an effective intervention.

Second, if effective, Purrble deployments could support professional clinical services at universities. One example of this would be Purrble as a complementary resource for those on waitlists. Given that waitlists are long in many countries: in the United Kingdom, for example, 54% of students reported waiting over 3 months to start treatment, and a further 12% reported waiting over a year [66]; additional support for those on waitlists is needed to aid well-being, help-seeking, and perceptions of care [67].

Third, Purrble could support emergent ER skills taught in traditional therapies, such as dialectical behavior therapy [68], serving as an additional support tool provided while on a waitlist, during or between therapeutic sessions, and potentially being of use to maintain skills once therapy is concluded. In particular, dialectical behavior therapy focuses on teaching skills, such as mindfulness, distress tolerance (distraction, reframing the moment, self-soothing, and problem-solving), interpersonal effectiveness, and ER, and seems aligned with the grounding and mindful interaction promoting effects of Purrble as reported by the students in this sample [47].

### Limitations

The following limitations of this study need to be considered. First, as an exploratory open trial, the size of the effects could have been affected by a range of time effects. For example, we would expect that the alignment of the study with term time could also lead to a reduction in anxiety scores, for example, due to a reduction of examination-induced anxiety at the end of the term. Additionally, we might see potential reduction-to-mean effects, as the sample was selected on the elevated need of anxiety symptoms at the start of term. Finally, it is possible that the data were also affected by COVID-19-driven cohort effects: at the time the study was conducted, the United Kingdom had recently ended a national lockdown due to COVID-19, which had detrimental effects on young people’s—and particularly students’—mental well-being [8]. It is likely that the students were at risk of heightened stress during the study, which might have further amplified the observed effects and perceived need.

Second, the study has not fully explored any gender effects in the acceptability or feasibility of Purrble. Initially, a large number of female students responded to the intervention advert, which prompted the selection of nonfemale responders and clinical need inclusion criteria. The initial disproportionate response from females may reflect a greater receptivity to the intervention concept, or potentially, this reflects a higher incidence of emotional distress, which is often presented as anxiety. For example, females (38%) present more commonly with clinically significant anxiety than males (20.3%) [10]. Alternatively, female participants are often overrepresented in psychological research [69]. As this trial was to assess the feasibility and acceptability of Purrble, a generalization sample across gender identities was selected. Alternatively, in future trials, there may be a benefit to maintaining the clinical threshold but removing gender limits.

Finally, additional mental health difficulties (eg, depression) or life factors (eg, grief) were not measured. Given that mental health difficulties are often comorbid, for example, 27.8% of a recent student sample reported both anxiety and depression [2], capturing such experiences is vital to understanding the impact and effectiveness of Purrble. Thus, it is not currently possible to examine in greater detail whether Purrble was useful for students dealing with these difficulties, which is discussed within the thematic analysis. It is also unclear whether the ER strategies induced by Purrble translate to these difficulties.

### Conclusions

Purrble appears to represent a feasible in situ intervention to aid ER within a university student population. In particular, Purrble was seen to be an acceptable tangible device that was useful in stressful and anxious moments, where students could downregulate their emotions. Moreover, its use was associated with a decrease in anxiety symptoms and an increase in ER competence among anxious college students. From this study, there are several research implications, such as exploring the impact of Purrble compared to a control group to better determine how Purrble is effective as an ER intervention and to what extent within highly anxious student populations. By using a longitudinal study design for this follow-up study, better evidence would be provided regarding the true impact of the intervention compared to this study, which aligned with the end of term time.

## Appendix

Multimedia Appendix 1 Optional daily survey questions.

Multimedia Appendix 2 Illustrative quotes.

## Abbreviations

DERS-18: Difficulties in Emotion Regulation Scale-18
ER: emotion regulation
GAD-7: Generalized Anxiety Disorder-7
TWEETS: Twente Engagement With eHealth Technologies Scale
