# Supplementary material for: Feasibility, Perceived Impact, and Acceptability of a Socially Assistive Robot to Support Emotion Regulation With Highly Anxious University Students: Mixed Methods Open Trial
Source: JMIR Ment Health. 2023 Oct 31;10:e46826. doi: 10.2196/46826 (PMC10646679; doi:10.2196/46826)
Supplement: Multimedia Appendix 1 [file mental_v10i1e46826_app1.docx]

# **DAILY EMAs**

[All the questions to be answered using sliders online, ideally on the phone]

1. **How was your day?**
   - Overall, how stressful was today for you?

*<not at all stressful> ← → <extremely stressful>*

- - Overall, how busy were you today with uni work?

*<not at all busy> ← → <extremely busy>*

- - Overall, how joyful were you today?

*<not at all joyful> ← → <extremely joyful>*

1. **How many times did you engage with Purrble today?**

*<not even once (0)> ← → <we were inseparable (more than 5 times)>*

- - If not even once — why? [checkbox]
    - didn't feel stressed [include optional open-ended textarea to expand on the answer]
    - didn't think it would help me today [include optional open-ended textarea to expand on the answer]
    - other [open-ended, text area]

1. **For how long would you say that you usually engaged with Purrble today?**

<minutes 0 ← → 10 or more>

1. **Did it usually make any difference to how you felt at the time?**

<not at all> ← → <very much>

- - If yes, then positive / negative impact? [checkbox]
  - Optional open-ended text to expand on the answer [textarea]
