# Supplementary material for: Feasibility, Perceived Impact, and Acceptability of a Socially Assistive Robot to Support Emotion Regulation With Highly Anxious University Students: Mixed Methods Open Trial
Source: JMIR Ment Health. 2023 Oct 31;10:e46826. doi: 10.2196/46826 (PMC10646679; doi:10.2196/46826)
Supplement: Multimedia Appendix 2 [file mental_v10i1e46826_app2.docx]

|  | | |
| --- | --- | --- |
| **Theme** | **Subtheme** | **Illustrative Quotes** |
| Primary uses for Purrble | Tool for anxiety and stress reduction | “*It really is very good at calming you down in stressful situations, like a hard tutorial or something, as long as it's a short duration.”* (R_25KK).  “*It is a good assistance to those with anxiety disorders who would otherwise not be able to mediate or relax.*” (R_3qCK). |
|  | Calming during night routine | “*Purrble has been most helpful in the evenings, giving me something to focus on that is not a screen, before I go to bed*” (R_OveS).  “*It has been quite comforting to sleep with Purrble in my bed, I tend to sleep better with stuffed animals in my bed*” (R_2CBa). |
|  | Prevention of mental health spiral | “I *found Purrble most useful when trying to calm down from minor episodes of anxiety to prevent spiralling into more severe anxiety (e.g., if I was worried about receiving some piece of work back, using the Purrble could prevent spiralling into more existential worries)*” (R_1dNd). |
| Appropriation mechanisms | Grounding | “*When I felt overwhelmed by the amount of work that I had to do, stroking Purrble helped me relax and be more centred in the present moment*” (R_2YX). |
|  | Mindfulness | “*Purrble works well in conjunction to breathing exercises as it acts as a physical reminder to engage (i.e. time breaths to purring) and can enhance this for me*” (R_2AH).  “*it is sometimes more useful to have a material aid (i.e. Purrble) - I think it to some extent overcomes the block of overthinking which can often interfere with mindfulness techniques.*” (R_3FU). |
|  | Self-stimulation | “*​​I'm quite a tactile person and I've often found that doing something with my hands could help calm me down. The Purrble fulfilled this role.*” (R_1dNd).  “*The action of stroking him rhythmically is very calming, and takes the focus away from the source of panic/stress*” (R_25vd). |
| Purrble is “just” a toy | Feelings of embarrassment | “I *have found Purrble not particularly useful to me during the day because I can't take it with me to the lab so I couldn't use it when I would have liked to*” (R_3Gkq).   “*self-conscious being an adult woman cuddling a robot*” (R_1K6H). |
|  | “If levels are too high” | “*knowing that it is merely a substitute and not an actual living being, when the day is particularly challenging, stressful or traumatising (due to grief) it doesn't help at all because it is stupid little toy”* (R_3Rz4).  “*Sometimes when I am already very stressed, even though I know I could try using him, I just feel too stressed and it is hard to make myself reach out for him*” (R_10ZQ). |
|  | Purrble-related anxiety | “*the rare/occasional times where Purrble's heartbeat/sounds trigger my overstimulation/anxiety*” (R_28Z2). |
| Empathetic response | Caring for something else helps one’s ability to self soothe | “*it made me feel quite parental and allowed me to get out of my own head for a minute*” (R_2CBa).  “*it felt as though i had to calm myself down in order to “help” the purrble calm down too. The responsibility helped*” (R_3PTu). |
|  | Guilt relating to “upsetting” Purrble | “*When I've been stressed or otherwise wanted to initiate interaction with Purrble, I've felt negative waking it up and causing its distress. Sometimes this has been enough to dissuade me from using my Purrble to help, or has initially made me feel worse rather than better*” (R_C85i). |
